# Adolescent sleep patterns, genetic predisposition, and risk of multiple sclerosis

**DOI:** 10.1093/sleep/zsae156

**Published:** 2024-07-08

**Authors:** Eva Johansson, Tomas Olsson, Pernilla Strid, Ingrid Kockum, Lars Alfredsson, Anna Karin Hedström

**Affiliations:** Department of clinical neuroscience, Karolinska Institutet, Stockholm, Sweden; Department of clinical neuroscience, Karolinska Institutet, Stockholm, Sweden; Department of Neurology, Karolinska University Hospital, Stockholm, Sweden; Department of clinical neuroscience, Karolinska Institutet, Stockholm, Sweden; Department of clinical neuroscience, Karolinska Institutet, Stockholm, Sweden; Department of clinical neuroscience, Karolinska Institutet, Stockholm, Sweden; Institute of Environmental Medicine, Karolinska Institutet, Stockholm, Sweden; Center for Occupational and Environmental Medicine, Region Stockholm, Sweden; Department of clinical neuroscience, Karolinska Institutet, Stockholm, Sweden; Department of Neurology, Karolinska University Hospital, Stockholm, Sweden

**Keywords:** multiple sclerosis, sleep duration, sleep quality, human leukocyte antigen

## Abstract

**Study Objectives:**

Shift work, insufficient sleep, and poor sleep quality at young age have been associated with increased risk of multiple sclerosis (MS). This study aimed to investigate the potential interaction between aspects of inadequate sleep (short sleep, phase shift, and poor sleep quality) during adolescence and HLA-DRB1*15:01 in relation to MS risk.

**Methods:**

We used a Swedish population-based case–control study (1253 cases and 1766 controls). Participants with different sleep patterns during adolescence and HLA-DRB1*15:01 status were compared regarding MS risk by calculating odds ratios with 95% confidence intervals (CI) using logistic regression models. Additive interaction between aspects of inadequate sleep and HLA-DRB1*15:01 status was assessed by calculating the attributable proportion due to interaction (AP) with 95% CI.

**Results:**

Short sleep duration (<7 hours/night) during adolescence acted synergistically with HLA-DRB1*15:01, increasing the risk of MS (AP 0.38, 95% CI: 0.01 to 0.75, *p* = .04). Similarly, subjective low sleep quality during adolescence interacted with HLA-DRB1*15:01 regarding risk of MS (AP 0.30, 95% CI: 0.06 to 0.56, *p* = .03), whereas phase shift did not significantly influence the risk of the disease, irrespective of HLA-DRB1*15:01 status.

**Conclusions:**

Our findings underscore the importance of addressing inadequate sleep during adolescence, particularly in the context of the HLA-DRB1*15:01 allele, as it appears to amplify the risk of subsequently developing MS.

**Statement of Significance**

This study reveals a significant interaction between inadequate sleep during adolescence and the HLA-DRB1*15:01 allele in increasing the risk of multiple sclerosis (MS). The findings highlight the synergistic effect of short sleep duration and poor sleep quality with genetic predisposition, emphasizing the critical role of sufficient and quality sleep in mitigating MS risk. These results underscore the potential for early lifestyle interventions, particularly sleep optimization, to reduce the risk of MS among genetically susceptible individuals.

Multiple sclerosis (MS) is a chronic immune-mediated disorder of the central nervous system, influenced by a combination of genetic and environmental factors, including Epstein-Barr virus infection, smoking, adolescent body mass index, sun exposure, and vitamin D. Among the genetic factors, the HLA-DRB1*15:01 allele stands out as the most significant and has been shown to interact with various environmental and lifestyle factors in MS [1–3].

Shift work has been linked to increased risk of MS in Nordic studies, particularly when such work commences at a young age [4, 5]. Given that irregular sleep–wake patterns and restricted sleep duration often result from shift work, we previously explored how specific aspects of sleep habits impact the occurrence of MS. Both restricted sleep and poor sleep quality were linked to increased MS risk, while the timing of sleep did not show a significant association [6]. Sleep deprivation and poor sleep quality affects immune pathways leading to a chronic inflammatory state [7], which could contribute to explain the associations.

Inadequate or disturbed sleep is a common issue among adolescents, in part due to physiologic, psychological, and social changes occurring during this life stage [8]. Since sleep patterns can be modified through lifestyle adjustments, it is important to further investigate their impact on MS risk. In the present study, our objective was to explore potential synergistic effect between aspects of inadequate sleep and presence of HLA-DRB1*15:01 allele in the context of MS risk.

## Methods

### Design and study population

We used a Swedish population-based case–control study, the Epidemiological Investigation of MS (EIMS), comprising the general population aged 16–70 years. Incident cases of MS were identified from neurology units in both hospitals and private clinics. The diagnosis was established based on the McDonald criteria by local neurologists [9, 10]. For each case, two controls were randomly selected from the national population register, matched to the cases based on age within 5-year age groups, sex, and residential area. The overall structure and methodology of this study have previously been described in greater detail elsewhere [3].

Information on lifestyle factors and various exposures was collected using a standardized questionnaire. Between April 2005 and March 2013, we received completed questionnaires from 2055 cases and 4518 controls, with response rates of 93% and 73%, respectively. In November 2013, we administered additional questions to all participants who had completed the original questionnaire during the abovementioned period. These supplementary questions included inquiries about sleep habits. Among the respondents, 1686 cases (82%) and 2982 controls (66%) provided answers to these supplementary questions. We excluded individuals with disease onset before the age of 20 years (225 cases and 308 controls) and those who were unable to answer the questions pertaining to sleep habits (110 cases and 305 controls). The inability to answer these questions were due to difficulties in remembering or having had irregular sleep patterns. Furthermore, all participants who participated in EIMS were requested to provide blood samples for genetic analyses. HLA genotypes were available for 1253 cases and 1766 controls, accounting for 61% and 39%, respectively, of those who initially completed the original questionnaire. The present study thus includes 1253 cases and 1766 controls (Supplementary Figure 1).

The study was approved by the Regional Ethical Review Board at the Karolinska Institute (2004/1-4:6) and was conducted in accordance with the ethical standards of the 1964 Declaration of Helsinki and its subsequent amendments. All participants provided informed consent prior to their involvement in the study.

### Definition of exposure variables

Given that previous research has predominantly linked shift work to increased risk of MS when the exposure occurs during early adulthood and considering that changes in sleep patterns may also result from MS itself, we focused on sleeping behaviors during the age period 15–19 years. To gather data on sleep, we used questions from the Karolinska Sleep Questionnaire, a validated instrument [11]. The questions were slightly modified to capture sleep habits across various age ranges (Supplementary Table 1).

#### Sleep duration.

Participants were requested to provide estimates of their usual bedtime and wake-up times on work or school and on weekends or free days for different age periods. These periods included ages 15–19, 20–29, 30–39, and 40 or older. To determine the average nightly sleep duration, we calculated it by dividing the total weekly sleep duration by seven, providing a per-night estimate. This method aimed to capture habitual sleep patterns over these age periods. Regarding the age period of 15–19 years, habitual sleep duration was dichotomized using 7 or fewer hours/night as cutoff.

#### Phase shift.

The change in sleep timing between work–schooldays and weekends–free days was calculated. Phase shift during the ages of 15–19 years was dichotomized using a cutoff of 3 hours.

#### Sleep quality.

For evaluating sleep quality, participants were asked to rate the quality of their sleep during different age periods using a 5-grade scale, ranging from very bad to very good. Sleep quality during the age period of 15–19 years was dichotomized into high quality (comprising rather good or very good) or low quality (comprising neither good nor bad, rather bad, or very bad).

### Genotyping

HLA alleles were identified at four-digit resolution. The genotyping process was conducted using the MS replication chip [12] and for HLA allele identification, HLA*IMP:02 imputation was employed [13]. Participants were categorized based on the presence or absence of specific HLA alleles that have previously been associated with risk of MS (HLA-DRB1*15:01, DRB1*03:01, DRB1*13:03, DRB1*08:01, A*02:01, B*44:02, B*38:01, B*55:01, DQA1*01:01, DQB1*03:02, and DQB1*03:01) [1, 2].

### Statistical analysis

We calculated correlations between habitual sleep duration, phase shift, and sleep quality using Pearson correlation coefficients with 95% confidence intervals (CI). Participants were categorized based on their habitual sleep duration (using a cutoff of 7 or fewer hours/night) and their HLA-DRB1*15:01 status. We compared the groups regarding the occurrence of MS by calculating odds ratios (OR) with 95% CI using logistic regression models. Furthermore, we assessed potential interactions on the additive scale, which is defined by deviation from additivity of effects, between short sleep duration (7 or fewer hours/night) and presence of DRB1*15:01 assessed by calculating the attributable proportion due to interaction (AP) with 95% CI. In a similar manner, participants were categorized based on HLA-DRB1*15:01 status and sleep quality. We then conducted analogous calculations to evaluate potential interactions.

All analyses were adjusted for age, sex, residential area, ancestry, smoking, infectious mononucleosis (IM), sun exposure, and for the following HLA alleles which have been independently associated with risk of MS: DRB1*03:01, DRB1*13:03, DRB1*08:01, B*44:02, B38:01, B44:02, DQA1*01:01, DQB1*03:02, and DQBI*03:01 [1, 2]. Ancestry was dichotomized into Swedish or non-Swedish origin. Smoking was categorized into current, past, or never smoking. IM was dichotomized into yes, no, or unknown. We constructed an index by summing the values to obtain a score between 3 and 12. The median value (6) was used to dichotomize sun exposure into low or high. The rationale for adjusting for these factors was that they are both associated with sleep patterns and the risk of MS. Since obesity may be a consequence of sleep-related factors, we did not include BMI in our final analyses. However, we conducted supplementary analyses further adjusted for BMI at age 20 years. Since sleep duration and sleep quality are correlated, we also investigated synergistic effects between short sleep duration and DRB1*15:01 status restricted to participants with high sleep quality. Similarly, we assessed interaction between low sleep quality and DRB1*15:01 status among participants who slept more than 7 hours/night. All analyses were conducted using Statistical Analysis System (SAS) version 9.4.

## Results

Our analyses were based on 1253 cases and 1766 controls. The mean age at disease onset among cases was 35.1 years (SD 10.5). Characteristics of cases and controls in the overall sample and by each aspect of sleep at age 15–19 years, are presented in Tables 1–3.

**Table 1.** Characteristics of Cases and Controls, in Total Sample and by Sleep Duration (Age Period 15–19 Years)

	Total		7 hours/night or less		>7 hours/night	
	Cases	Controls	Cases	Controls	Cases	Controls
*N*	1253	1766	72	83	1181	1683
Women, *n* (%)	943 (75)	1328 (75)	47 (65)	55 (66)	896 (76)	1273 (76)
Swedish, *n* (%)	1012 (81)	1413 (80)	51 (71)	66 (80)	961 (81)	1347 (80)
Phase shift, mean no. of hours (SD)	1.2 (1.0)	1.2 (1.0)	1.2 (1.3)	1.2 (1.0)	1.2 (1.3)	1.2 (1.0)
Sleep quality, mean (SD)	4.3 (0.9)	4.4 (0.8)	3.6 (1.3)	3.7 (1.2)	4.3 (0.8)	4.4 (0.8)
Smoking, *n* (%)	640 (51)	759 (43)	49 (68)	51 (61)	591 (50)	708 (42)
Infectious mononucleosis, *n* (%)	226 (18)	197 (11)	13 (18)	14 (17)	213 (18)	183 (11)
Sun exposure (SD)	6.3 (1.8)	6.6 (1.9)	6.0 (2.1)	6.4 (1.8)	6.3 (1.8)	6.6 (1.9)
Mean adolescent BMI, kg/m (SD)	22.3 (3.7)	21.7 (3.0)	22.5 (4.1)	22.7 (3.8)	22.3 (3.7)	21.7 (2.9)
HLA-DRB1*15:01 carrier, *n* (%)	691 (55)	207 (29)	37 (51)	17 (20)	654 (55)	490 (29)
Age at disease onset (SD)	35.1 (10.5)		35.9 (12.2)		35.0 (10.4)	

**Table 2.** Characteristics of Cases and Controls, by Phase Shift (Age Period 15–19 Years)

	<3 hours		3 hours or more	
	Cases	Controls	Cases	Controls
*N*	104	140	1149	1626
Women, *n* (%)	82 (79)	108 (77)	861 (75)	1220 (75)
Swedish, *n* (%)	84 (81)	113 (81)	928 (81)	1300 (80)
Sleep duration, hours/night (SD)	8.5 (0.9)	8.6 (0.8)	8.3 (0.8)	8.3 (0.9)
Sleep quality, mean value (SD)	4.3 (0.8)	4.4 (0.9)	4.3 (0.9)	4.4 (0.8)
Smoking, *n* (%)	67 (64)	70 (50)	573 (50)	689 (42)
Infectious mononucleosis, *n* (%)	24 (23)	27 (19)	202 (18)	170 (10)
Sun exposure (SD)	6.3 (1.8)	6.7 (2.0)	6.3 (1.8)	6.6 (1.9)
Mean adolescent BMI, kg/m (SD)	22.1 (4.1)	21.9 (3.6)	22.6 (3.7)	21.7 (2.9)
HLA-DRB1*15:01 carrier, *n* (%)	61 (59)	45 (32)	630 (55)	462 (28)
Age at disease onset (SD)	35.0 (10.0)		35.1 (10.5)	

Phase shift = difference in the timing of sleep between work–schooldays and days off.

**Table 3.** Characteristics of Cases and Controls, by Sleep Quality (Age Period 15–19 Years)

	Low sleep quality (< 5)		High sleep quality (5)	
	Cases	Controls	Cases	Controls
*N*	192	182	1061	1584
Women, *n* (%)	142 (74)	143 (79)	801 (75)	1185 (75)
Swedish, *n* (%)	154 (80)	139 (76)	858 (81)	1274 (80)
Phase shift, mean no. of hours (SD)	1.1 (1.0)	1.3 (1.2)	1.2 (1.2)	1.2 (1.0)
Sleep duration, hours/night (SD)	8.1 (1.1)	8.0 (1.1)	8.6 (0.8)	8.6 (0.8)
Smoking, *n* (%)	112 (58)	81 (45)	528 (50)	678 (43)
Infectious mononucleosis, *n* (%)	37 (19)	18 (10)	189 (18)	179 (11)
Sun exposure (SD)	6.1 (1.9)	6.8 (2.0)	6.3 (1.8)	6.5 (1.9)
Mean adolescent BMI, kg/m (SD)	22.7 (4.0)	22.3 (3.4)	22.3 (3.6)	21.7 (2.9)
HLA-DRB1*15:01 carrier, *n* (%)	103 (54)	48 (26)	588 (55)	459 (29)
Age at disease onset (SD)	33.6 (11.4)		35.3 (10.3)	

Sleep quality was assessed on a 5-grade scale (1 = lowest quality, 5 = highest quality).

Habitual sleep duration and sleep quality were significantly correlated (*r* = .3, *p* < .0001 among both cases and controls), whereas phase shift did not show a significant correlation with either sleep duration or sleep quality. In the total sample, the OR of MS was 1.45 (95% CI: 1.05 to 1.85) among participants with short sleep duration, compared with those who slept more than 7 hours/night and 1.60 (95% CI: 1.32 to 1.97) among those with low sleep quality, compared to those who reported high sleep quality. A shift in the timing of sleep of more than 3 hours between work–schooldays and days off was not associated with risk of MS (OR 0.97, 95% CI: 0.87to 1.15).

### Interactions between aspects of sleep and HLA-DRB1*15:01

Compared with HLA-DRB1*15:01 negative individuals with a sleep duration of more than 7 hours/night, the adjusted OR of MS was 6.01 (95% CI: 3.26 to 11.10) among HLA-DRB1*15:01 carriers with short habitual sleep duration. The AP between HLA-DRB1*15:01 and short sleep duration with respect to MS was 0.38 (95% CI: 0.01 to 0.75), *p* = .04 (Table 4). The interaction remained significant when the analysis was limited to participants who reported high sleep quality (AP 0.34, 95% CI: 0.09 to 0.85, *p* = .05).

**Table 4.** OR of MS With 95% CI for Participants With Different HLA-DRB1*15:01 Status and Habitual Sleep Duration

DRB1*15:01	Hours/night	ca/co^a^	OR (95% C)^b^	OR (95% CI)^c^	AP (95% CI)^c^, *p* value
-	>7	519/1169	1.0 (reference)	1.0 (reference)	
-	7 or less	32/61	1.19 (0.78 to 1.81)	1.21 (0.81 to 1.86)	
+	>7	645/482	3.03 (2.59 to 3.54)	3.47 (2.84 to 4.24)	
+	7 or less	32/15	4.95 (2.76 to 8.88)	6.01 (3.26 to 11.1)	0.38 (0.01 to 0.75), .04

^a^number of exposed cases and controls;

^b^adjusted for age, sex, residential area, and ancestry;

^c^adjusted for age, sex, residential area, ancestry, smoking, infectious mononucleosis, sun exposure, phase shift, sleep quality, HLA-DRB1*03:01, DRB1*13:03, DRB1*08:01, A*02:01, B*44:02, B*38:01, B*55:01, DQA1*01:01, DQB1*03:02, and DQB1*03:01.

Subjective poor sleep quality among HLA-DRB1*15:01 carriers rendered an OR of 5.80 (95% CI: 3.94 to 8.54), compared to HLA-DRB1*15:01 negative individuals who rated their sleep quality as high. The AP between poor sleep quality and HLA-DRB1*15:01 was 0.30 (95% CI: 0.06 to 0.56), *p* = .03 (Table 5). The interaction remained significant when the analysis was based on only participants who slept more than 7 hours/night (AP 0.28, 95% CI: 0.06 to 0.58), *p* = .05.

**Table 5.** OR of MS With 95% CI for Participants With Different HLA-DRB1*15:01 Status and Sleep Quality

DRB1*15:01	Sleep quality	ca/co^a^	OR (95% C)^b^	OR (95% CI)^c^	AP (95% CI)^c^, *p* value
−	5	473/1125	1.0 (reference)	1.0 (reference)	
−	<5	89/134	1.56 (1.17 to 2.08)	1.56 (1.16 to 2.10)	
+	5	588/459	3.06 (2.60 to 3.60)	3.49 (2.84 to 4.29)	
+	<5	103/48	5.03 (3.51 to 7.21)	5.80 (3.94 to 8.54)	0.30 (0.06 to 0.56), .03

^a^number of exposed cases and controls;

^b^adjusted for age, sex, residential area, and ancestry;

^c^adjusted for age, sex, residential area, ancestry, smoking, infectious mononucleosis, sun exposure, sleep duration, phase shift, HLA-DRB1*03:01, DRB1*13:03, DRB1*08:01, A*02:01, B*44:02, B*38:01, B*55:01, DQA1*01:01, DQB1*03:02, and DQB1*03:01.

There was no sign of synergistic effects between phase shift and HLA-DRB1*15:01 regarding risk of MS (Table 6). All results remained consistent when the analyses were further adjusted for at age 20 years.

**Table 6.** OR of MS With 95% CI for Participants With Different HLA-DRB1*15:01 Status and Phase Shift Between Work/School Days and Free Days

DRB1*15:01	Phase shift (hours)	ca/co^a^	OR (95% C)^b^	OR (95% CI)^c^	AP (95% CI)^c^, *p* value
−	<3	519/1164	1.0 (reference)	1.0 (reference)	
−	3 or more	43/95	1.00 (0.69 to 1.46)	1.04 (0.71 to 1.52)	
+	<3	630/462	3.07 (2.62 to 3.60)	3.52 (2.87 to 4.31)	
+	3 or more	61/45	3.03 (2.03 to 4.52)	3.59 (2.34 to 5.50)	0.02 (−0.42 to 0.43), .97

^a^number of exposed cases and controls;

^b^adjusted for age, sex, residential area, and ancestry;

^c^adjusted for age, sex, residential area, ancestry, smoking, infectious mononucleosis, sun exposure, sleep duration, sleep quality, HLA-DRB1*03:01, DRB1*13:03, DRB1*08:01, A*02:01, B*44:02, B*38:01, B*55:01, DQA1*01:01, DQB1*03:02, and DQB1*03:01.

## Discussion

Both insufficient sleep and poor sleep quality appear to interact with the HLA-DRB1*15:01 allele, increasing the risk of subsequently developing MS. Irrespective of HLA-DRB1*15:01 status, a shift in timing of sleep was not associated with risk of MS.

Inadequate sleep affects immune functions through various mechanisms, such as elevated production of proinflammatory markers, systemic inflammation, and immune dysfunction [7]. Additionally, sleep deprivation may contribute to low-grade neuroinflammation, oxidative stress, and disruption of the blood-brain-barrier [14–16]. Additionally, during periods of sleep deprivation, circulating levels of melatonin, known to have neuroprotective and anti-inflammatory properties, tend to decrease as a protective response against the concurrent increase in oxidative stress [17]. We hypothesize that the combination of genetic susceptibility and disrupted sleep may enhance inflammatory processes and immune dysregulation, thereby increasing the risk of MS.

Insufficient or poor sleep adversely affects the body’s response to viral infections [18]. Epstein-Barr virus has been identified as a critical factor in the development of MS and increasing evidence suggest that the latent infection remains inadequately controlled in individuals with MS [19]. Viral reactivation can be triggered by various stressors, whether immune, cellular, or psychological in nature [20]. The compromised ability to manage viral infections due to insufficient sleep may also contribute to explain the association between inadequate sleep and increased risk of MS. Our findings underscore the complexity of the disease’s etiology, emphasizing the need for further exploration into the interplay between genetics, immune function, and sleep patterns in the context of MS.

EIMS was designed as a population-based, case–control study where information on lifestyle factors was gathered retrospectively. Given that changes in sleep patterns may follow neurodegeneration, our study aimed to explore the potential interaction between HLA-DRB1*15:01 and sleep habits during the age period 15–19 years regarding subsequent MS risk. Our questions about sleep were rather general and we did not have more detailed information on sleep disorders. Additionally, we had no objective data on sleep. Since individuals with MS frequently encounter sleep disorders and fatigue [21], it is plausible that cases may recall their previous sleep habits differently compared to controls. However, our finding of an interaction between short sleep/low sleep quality and HLA-DRB1*15:01 strengthens the evidence of inadequate sleep as a risk factor for MS. It appears unlikely that bias would substantially impact on our findings, since any potential bias would depend both on recall of sleep patterns and HLA genotype. Additionally, the prior association observed between shift work and MS risk [4, 5] lends further support to our findings regarding the impact of short sleep/low sleep quality on the subsequent development of the disease. Respondents are unlikely to experience significant memory errors when answering questions about shift work. However, while we have taken steps to minimize bias, we cannot entirely eliminate the possibility of recall bias in our study.

The questions regarding sleep habits were answered by 82% of the cases and 66% of the controls, which raises concerns about potential selection bias. However, there were no differences observed in terms of age, sex, or smoking habits between those who participated in EIMS complementary questionnaire and those who did not. Moreover, the prevalence of lifestyle factors, such as smoking, among the controls was consistent with that of the general population [22]. Therefore, we are confident that our findings are not significantly influenced by selection bias.

Maintaining healthy sleep patterns is essential for overall immune health. Resent research has highlighted associations between excessive social media use and disrupted sleep patterns, particularly among adolescents. The pervasive availability of technology and internet access at all hours has contributing to insufficient sleep becoming a public health concern among this age group [23–28]. It underscores the significance of educational interventions aimed at adolescents and their parents, emphasizing the adverse health effects of insufficient sleep.

Future studies should consider incorporating validated sleep questionnaires with more detailed information on sleep quality and potential sleep disorders. Given the relatively low prevalence of MS, we recognize that implementing objective sleep measures such as polysomnography would be impractical and expensive. However, combining validated sleep questionnaires with wearable technology for continuous sleep monitoring on large scale could further enhance the depth and accuracy of sleep assessments, which would be important in understanding the role of sleep in MS development more comprehensively.

In conclusion, our findings underscore the importance of addressing inadequate sleep during adolescence, particularly in the context of the HLA-DRB1*15:01 allele, as it appears to amplify the risk of subsequently developing MS.

## Supplementary material

Supplementary material is available at *SLEEP* online.

zsae156_suppl_Supplementary_Materials

## Data Availability

Anonymized data underlying this article will be shared on reasonable request from any qualified investigator that wants to analyze questions that are related to the published article.
