# Supplementary material for: Adolescent sleep patterns, genetic predisposition, and risk of multiple sclerosis
Source: Sleep. 2024 Jul 8;47(10):zsae156. doi: 10.1093/sleep/zsae156 (PMC11467049; doi:10.1093/sleep/zsae156)
Supplement: zsae156_suppl_Supplementary_Materials [file zsae156_suppl_supplementary_materials.zip › SLEEP-2024-0285.R1_SupportingInformationFile.docx]

**Adolescent sleep patterns, genetic predisposition, and risk of multiple sclerosis**

Eva Johansson^1^, Tomas Olsson^1-2^, Strid P^1^, Kockum I^1^, Lars Alfredsson^1,3-4^, Anna Karin Hedström^1-2^.

^1^Department of clinical neuroscience, Karolinska Institutet, Stockholm, Sweden.
^2^Karolinska University Hospital, Stockholm, Sweden.
^3^Institute of Environmental Medicine, Karolinska Institutet, Stockholm, Sweden. ^4^Center for Occupational and Environmental Medicine, Region Stockholm, Stockholm, Sweden.

Corresponding author details
Anna Karin Hedström

Department of clinical neuroscience, Visionsgatan 18, L8, 17176, Stockholm, Karolinska Institutet

Mobile phone: +0046762736426

E-mail address: [anna.hedstrom@ki.se](mailto:anna.hedstrom@ki.se)

Supplementary information 1. **Questions from EIMS**

**Sleep duration**

At which time do or did you normally go to sleep during work or schooldays?

15-19 years Clock time:

20-29 years Clock time:

30-39 years Clock time:

40 years or older Clock time:

At which time do or did you normally wake up during work or schooldays?

15-19 years Clock time:

20-29 years Clock time:

30-39 years Clock time:

40 years or older Clock time:

At which time do or did you normally go to sleep during weekends or days off?

15-19 years Clock time:

20-29 years Clock time:

30-39 years Clock time:

40 years or older Clock time:

At which time do or did you normally wake up during weekends or days off?

15-19 years Clock time:

20-29 years Clock time:

30-39 years Clock time:

40 years or older Clock time:

**Sleep quality**

Generally, how would you describe your sleep?

very bad, rather bad, neither good nor bad, rather good, very good

**Smoking**

If you **do not smoke**, and **have never smoked**, put a cross in the box and proceed to the next section.

If you **smoke regularly**, or **have smoked regularly**, put a cross in the box.
Below, specify the time-period/time-periods and amount.

Try to specify the average amount. Nb! The number is specified **per day**.

From age To age Number of cigarettes per day
 Number of cigars/cheroots per day Number of pipe fills per day

If you **smoke non-regularly**, for example at parties, or **have smoked non-regularly**, put a cross in the box. Below, specify the time-period/time-periods and amount.

Try to specify the average amount. Nb! The number is specified **per week**.

From age To age Number of cigarettes per week
 Number of cigars/cheroots per week Number of pipe fills per week

**Sun exposure**

If the weather is sunny, how often do you usually sunbathe?

Never A few times a month A few times a week Daily

How often, during the last 5 years, have you visited a country that is sunnier than Sweden?

Never More seldom Once a year More than once a year

How often, during the last 5 years, have you used a sunbed?

Never A few times a year Once a month Once a week

**Infectious mononucleosis**

Infectious diseases that usually occur during childhood or youth

Have you had these infectious diseases?

1. Measles No Yes Don’t know If “yes”, specify at what age you think you had the disease:

2. Mumps

3. Whooping cough

4. Chickenpox

5. Glandular fever

6. Other severe/long-lasting infection
If “yes”, specify what infection


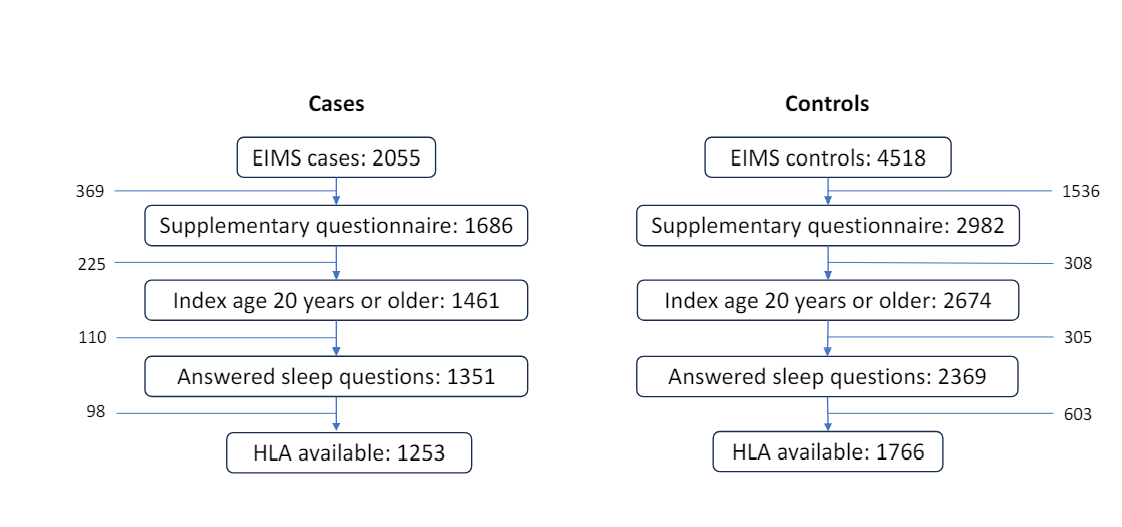


Supplementary figure 1. Number of cases and controls included in the study.
