# Supplementary figures and images for: Adolescent sleep patterns, genetic predisposition, and risk of multiple sclerosis
Source: Sleep. 2024 Jul 8;47(10):zsae156. doi: 10.1093/sleep/zsae156 (PMC11467049; doi:10.1093/sleep/zsae156)

## Cases

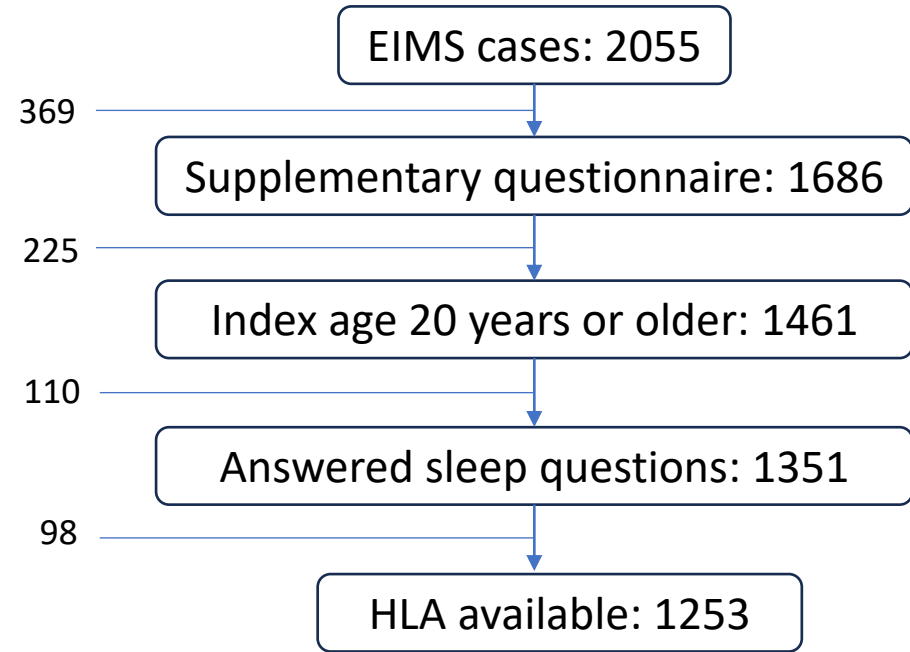

## Controls

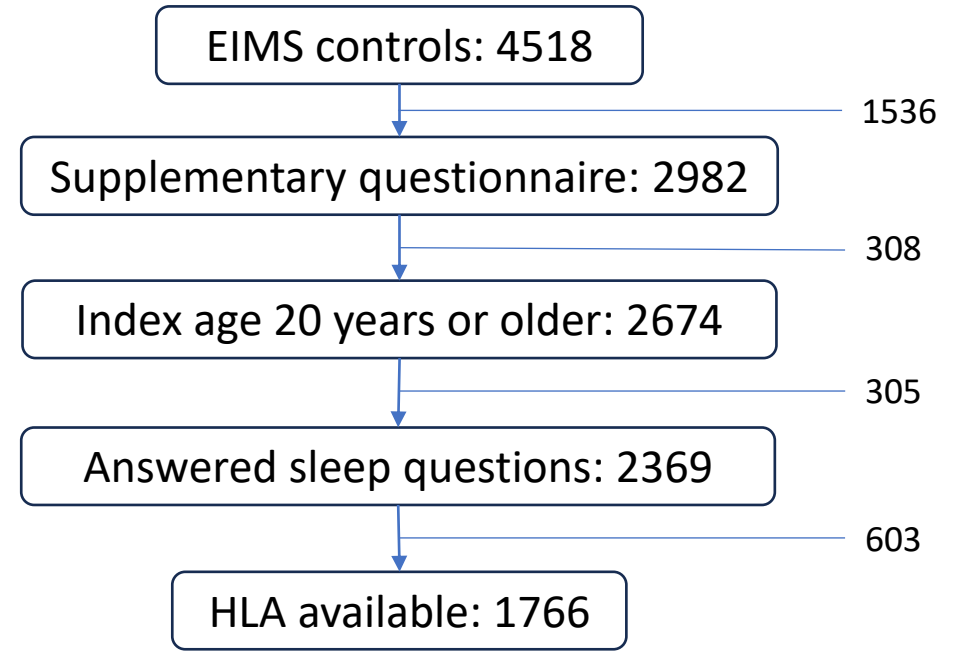

Supplement: zsae156_suppl_Supplementary_Materials [file zsae156_suppl_supplementary_materials.zip › Figure 1.pdf]
